# No disadvantages for women in acute stroke care in Germany: an analysis of access to stroke treatment services in Germany from 2017 to 2022

**DOI:** 10.1186/s42466-025-00365-4

**Published:** 2025-02-20

**Authors:** Matthias N. Ungerer, Dirk Bartig, Christine Tunkl, Daniel Richter, Aristeidis Katsanos, Christos Krogias, Werner Hacke, Christoph Gumbinger

**Affiliations:** 1 Department of Neurology, Heidelberg University Hospital, Heidelberg, Germany; 2 DRG Market, Osnabrück, Germany; 3 Department of Neurology, Evangelisches Krankenhaus Herne, Academic Teaching Hospital of the Ruhr University Bochum, Herne, Germany; 4 Division of Neurology, McMaster University and Population Health Research Institute, Hamilton, ON Canada

**Keywords:** Sex characteristics, Ischemic stroke

## Abstract

**Background:**

Several publications have raised concerns that female stroke patients may be at a disadvantage when accessing stroke treatment services. These publications have found significant regional differences in the provision of stroke treatment to male and female patients. In this study, we provide current nationwide data on stroke management differences between men and women in Germany.

**Methods:**

This large retrospective cohort study used national datasets from the German Federal Statistical Office for 2017–2022. We examined differences between female and male stroke patients in terms of case volume, intravenous thrombolysis (IVT), mechanical thrombectomy (MTE), stroke unit (SU) treatment, intrahospital mortality, and prevalence of atrial fibrillation (AF).

**Results:**

Data from more than 1.3 million hospitalised stroke patients were included. Forty-seven percent of the patients were female. Female patients were older and more frequently ≥ 80 years old (50.3% versus 29.4%). Rates of IVT (16.3% versus 16.3%) were similar for both sexes but higher in females when adjusted for age. MTE rates (8.2% versus 6.3%) were consistently higher in female patients across all age groups. Female patients had higher rates of intrahospital mortality (9.1% versus 6.2%), and admission to SUs (73.6% versus 76.0%) was less common. Treatment rates in intensive care units were similar (10.6% versus 10.5%). AF, a surrogate for embolic (and more severe) strokes, was more prevalent in females (32.6% versus 25.4%).

**Conclusions:**

We found no evidence that female stroke patients in Germany face any disadvantage in accessing stroke treatment services. Acute stroke treatment rates were generally similar or higher when compared to males. Higher intrahospital mortality and lower SU rates were attributed to greater age, comorbidities, and stroke severity. However, the differences were not fully explained when adjusting for AF and age. Further research is needed on sex differences in stroke mechanisms and outcomes.

**Supplementary Information:**

The online version contains supplementary material available at 10.1186/s42466-025-00365-4.

## Introduction

The treatment of ischemic stroke has significantly improved over the past few decades. This is mainly due to the widespread availability of specialised stroke unit care and an increased use of acute recanalisation therapies, such as intravenous thrombolysis (IVT) and mechanical thrombectomy (MTE), which have led to a reduction in stroke morbidity and mortality rates. The current literature highlights persistent disparities in access to acute stroke care due to age, sex, ethnicity, socioeconomic status, and geography [8, 19, 26]. Sex is an important factor that has been associated with poor access to stroke treatment services. Female patients have been described globally as receiving fewer interventional treatments, and as having consistently poorer outcomes after stroke both in terms of overall mortality and functional outcomes [23]. Considerable regional sex differences in IVT rates were also evident in a meta-analysis of 24 studies drawing data from 2008–2018 with female patients having a 13% lower odds of receiving IVT, particularly in European and US-based studies, while no treatment differences were reported from Asia [24, 27]. Although increasing IVT rates have narrowed the gap in recent years, the disparity has persisted according to a more recent study from Denmark [17]. Another meta-analysis suggested similar MTE rates for females globally, with higher rates for females being reported in Europe [21]. A recent study based on large datasets from the United States also reported higher MTE rates in females [16]. Data previously published from German national datasets have consistently shown that female stroke patients received interventional treatments at similar or even higher rates than their male counterparts [4, 29]. This is partly due to a dense network of certified stroke units providing excellent access to comprehensive stroke care in Germany [1].

Despite extensive literature, regional sex differences in access to recanalisation treatments remain controversial [24]. Particularly in the field of MTE, rapid increases in treatment rates and the introduction of novel treatment concepts and techniques make regular reports on current data imperative for valid tracking of the progress made in this dynamic field. In the present study, we aimed to examine current data from large national datasets from the German Federal Statistical Office from 2017 to 2022 to report on trends in sex differences in access to stroke treatments in Germany.

## Methods

### Study design, setting, and data source

We conducted a retrospective cohort study in Germany (84.7 million habitants [3], central Europe) using national datasets based on German Diagnosis-Related Groups (G-DRG), covering a period from 2017 to 2022. Data collection was performed by the German Federal Statistical Office and datasets were obtained from the Destatis database (© Statistisches Bundesamt, 2024).

The legal basis for data collection was Sect. 21 para. 3 sentence 1 no. 4 of the Hospital Reimbursement Act (KHEntgG), in conjunction with Sect. 28 para. 4 of the Hospital Financing Act (KHG) and the Federal Statistics Act (BStatG) in the version applicable in the reporting year (https://www.gesetze-im-internet.de).

Extensive quality assurance measures are carried out by both the Institute for the Hospital Remuneration System (InEK) and the Federal Statistical Office (Destatis) in the context of a systematic and multi-stage data check (‘error procedure’) during data acceptance by the DRG data centre, as well as by performing complex plausibility and conformity checks during data preparation. Detailed clinical scores, such as patients’ NIHSS and mRS scores, as well as follow-up data after discharge from the hospital, are not part of the DRG data. The Destatis database includes patients with permanent residence in Germany and therefore may show slight differences compared to other datasets (the difference in total case numbers between the Destatis and InEK databases is < 0.1%). We used data from 2017 to 2022 in our analysis since data for the year 2023 have not yet been published in the Destatis database.

Data on treatment in intensive care units (ICUs) and in early rehabilitation facilities for the reporting years 2020 to 2022 were extracted from the InEK DatenBrowser [14]. All hospitals in Germany are mandated by law (Sect. 21 of the Hospital Remuneration Act) to report all billing data to InEK annually. InEK checks the completeness and plausibility of the data records and then transmits them to Destatis. Destatis then subjects these data to further systematic checking protocols (e.g. to eliminate duplicate data).

The analysis and reporting conducted in the present study adhered to the STROBE guidelines.

### Eligibility criteria

We included all patients admitted with the main diagnosis of ischemic stroke (ICD-10 code I63.-). Patients with a main diagnosis of I64.- (stroke, not specified) or I67.- (other cerebrovascular diseases) were not included. Further exclusion criteria were missing documentation or documentation of a biological sex other than male or female. To exclude double counting when determining the population with a main diagnosis of ICD I63, only the cases with first admissions were included (excluding cases with discharge code 06; transfer to another hospital). The case numbers for all treatment subgroups (IVT, MTE, SU, etc.) were determined without this discrimination.

### Variables

The study examined patient characteristics, such as age, sex, and comorbidity with atrial fibrillation (AF), as well as data on intrahospital mortality, IVT rates, MTE rates, access to stroke unit (SU) care, and admission to intensive care units (ICUs). Patients were grouped according to their age and reported sex throughout the manuscript. We assumed that the reported patient sex corresponded to the biological sex of the patients. Age was compared between groups both as means and by age category (< 50; 50–59; 60–69; 70–79; 80–89; ≥ 90) for specific variables between both groups in data generated from the Destatis database. In the case of treatment in ICUs, where data were obtained from the InEK database, we used the non-modifiable predefined age groups (< 50; 50–54; 55–59; 60–64; 65–74; 75–79; ≥ 80) of the database. Patients diagnosed with AF were identified using the ICD-10 code I48.- in combination with the main diagnosis I63.-. Patients who received IVT or MTE were identified using the procedural codes OPS 8–020.8 or OPS 8–836.80, respectively, in combination with the main diagnosis I63.-.

### Statistical analysis

Standard descriptive statistics were used to describe the data. Data on female stroke patients were consistently mentioned first throughout the manuscript whenever comparisons between female and male patients were made. Differences in frequencies were calculated using the chi-square test, and differences between continuous variables were calculated using the independent T-test. We used binary regression analysis to calculate odds ratios (ORs) adjusted for age categories in the comparison between female and male patients for pooled data from 2017 to 2022. Analyses of pooled data were corroborated with sensitivity analysis of individual yearly data. We also performed two subgroup analyses for age-adjusted ORs to estimate the association between AF (as a surrogate for embolic stroke) and intrahospital mortality and MTE rates. Percentages were rounded to one decimal point. P-values and ORs were rounded to two decimal points. A *p*-value ≤ 0.05 was considered statistically significant. The generation of figures and statistical analyses were performed using IBM® SPSS® Statistics Version 29.

## Results

**Fig. 1:**
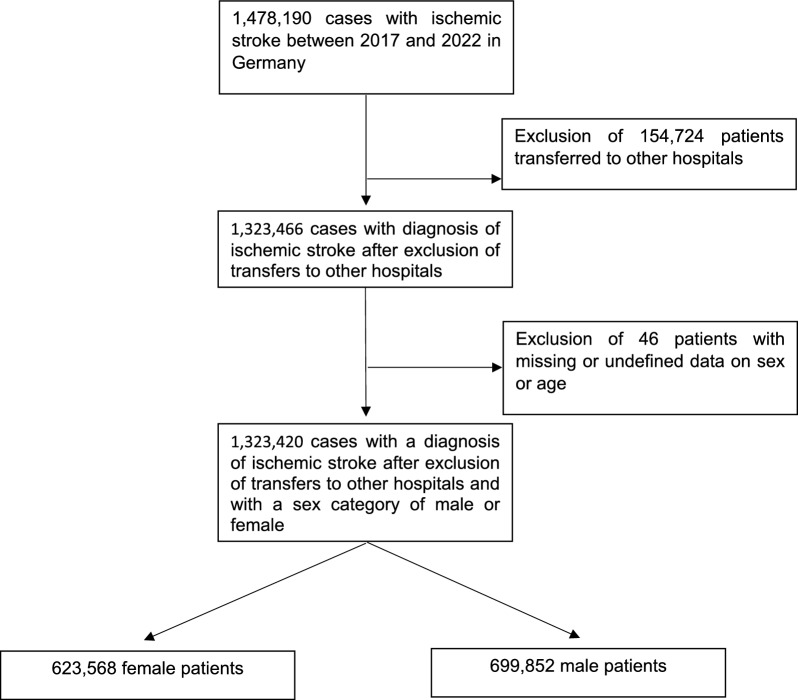
Flowchart showing the exclusion of cases

A total of 1,478,190 cases were registered with ischemic stroke (ICD-10 I63.-) as their main diagnosis between 2017 and 2022. To avoid double inclusion, 154,724 cases were excluded from the analysis due to transfer to another hospital. A further 46 patients were classified as unknown sex or as a sex category other than male or female and were also excluded from this analysis (see Fig. 1). Of all 1,323,420 stroke patients included in our analysis in the period of 2017–2022, 47.1% were female. The percentage of female patients decreased consistently every year, from 47.8% in 2017 to 46.5% in 2022. The mean age was 77.1 ± 5.4 years in female stroke patients compared to 71.5 ± 4.5 in male stroke patients. From 2017 to 2022, 39.2% of all stroke patients were 80 years or older, with female stroke patients consistently having a higher percentage of patients aged 80 or older (50.3% versus 29.4%; *p* < 0.01). Details of comparisons of case numbers broken down by sex are available in Table 1, while Table 2 shows an overview of ORs for females to receive stroke treatment services or to experience intrahospital mortality adjusted for age categories.

**Table 1:** Comparison of case numbers according to sex (2017–2022)

Item	Female	Male	Test	*p*-value
Cases (n)	623,568	699,852		
Age (mean, SD)	77.1 ± 5.4	71.5 ± 4.5	t-test	< 0.01
Age ≥ 80 years (n, % of column)	313,400 (50.3%)	205,388 (29.35%)	chi-square test	< 0.01
IVT (n, % of column)	101,767 (16.3%)	114,041 (16.3%)	chi-square test	0.696
MTE rate (n, % of column)	51,364 (8.2%)	44,278 (6.3%)	chi-square test	< 0.01
SU rate (n, % of column)	459,125 (73.6%)	531,921 (76.0%)	chi-square test	< 0.01
ICU rate (n, % of column)*	31,993 (10.6%)	36,022 (10.5%)	chi-square test	0.115
Early rehabilitation without SU or ICU (n, % of column)*	32,124 (10.7%)	29,273 (8.5%)	chi-square test	< 0.01
Intrahospital mortality rate (n, % of column)	56,739 (9.1%)	43,675 (6.2%)	chi-square test	< 0.01
AF rate (n, % of column)	202,961 (32.6%)	178,017 (25.4%)	chi-square test	< 0.01
Sensitivity analysis: Intrahospital mortality rate in AF (n, % of AF)	27,789 (13.7%)	18,993 (10.7%)	chi-square test	< 0.01
Sensitivity analysis: MTE rate in AF (n, % of AF)	26,613 (13.1%)	17,400 (9.8%)	chi-square test	< 0.01

*AF*, Atrial fibrillation; *SD*, Standard deviation; *ICU*, Intensive care unit; *IVT*, Intravenous thrombolysis; *MTE*, Mechanical thrombectomy; *SU*, Stroke unit

*InEK database (data from 2020–2022)

**Table 2:** Odds Ratios for female versus male stroke patients adjusted for age categories in binary regression analysis (2017–2022)

Item	Odds ratio	Lower CI	Upper CI	*p*-value
IVT rate	1.019	1.009	1.029	< 0.01
MTE rate	1.344	1.326	1.363	< 0.01
Intrahospital mortality rate	1.096	1.081	1.111	< 0.01
AF rate	1.045	1.036	1.053	< 0.01
SU rate	0.923	0.916	0.931	< 0.01
ICU rate*	1.032	1.015	1.049	< 0.01
Early rehabilitation without SU or ICU*	1.103	1.085	1.123	< 0.01
Sensitivity analysis: Intrahospital mortality rate in AF	1.101	1.079	1.123	< 0.01
Sensitivity analysis: MTE rate in AF	1.506	1.475	1.538	< 0.01

*AF*, Atrial fibrillation; *SD*, Standard deviation; *ICU*, Intensive care unit; *IVT*, Intravenous thrombolysis; *MTE*, Mechanical thrombectomy; *SU*, Stroke unit

*InEK database (data from 2020–2022)

### Comparison of stroke service treatment rates

**Fig. 2:**
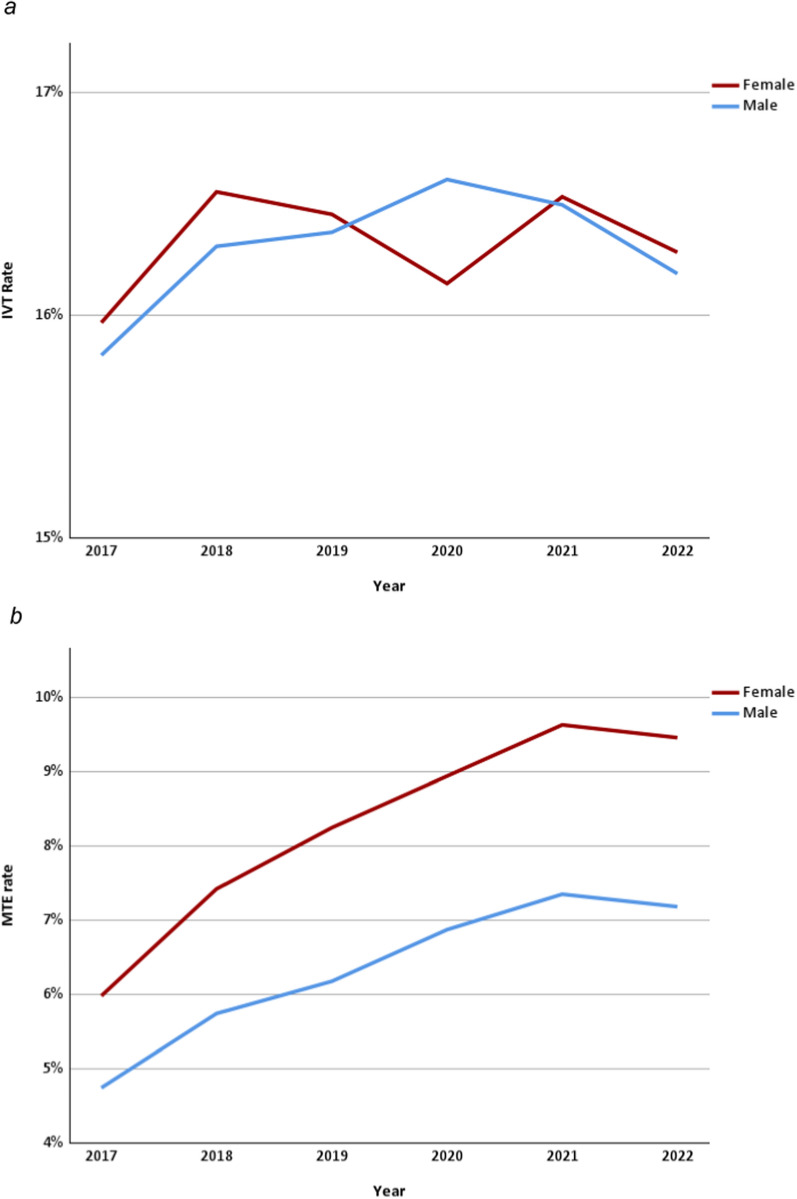
**a** IVT rates according to sex from 2017 to 2022. IVT, intravenous thrombolysis. **b** MTE rates according to sex from 2017 to 2022. MTE, mechanical thrombectomy

**Fig. 3:**
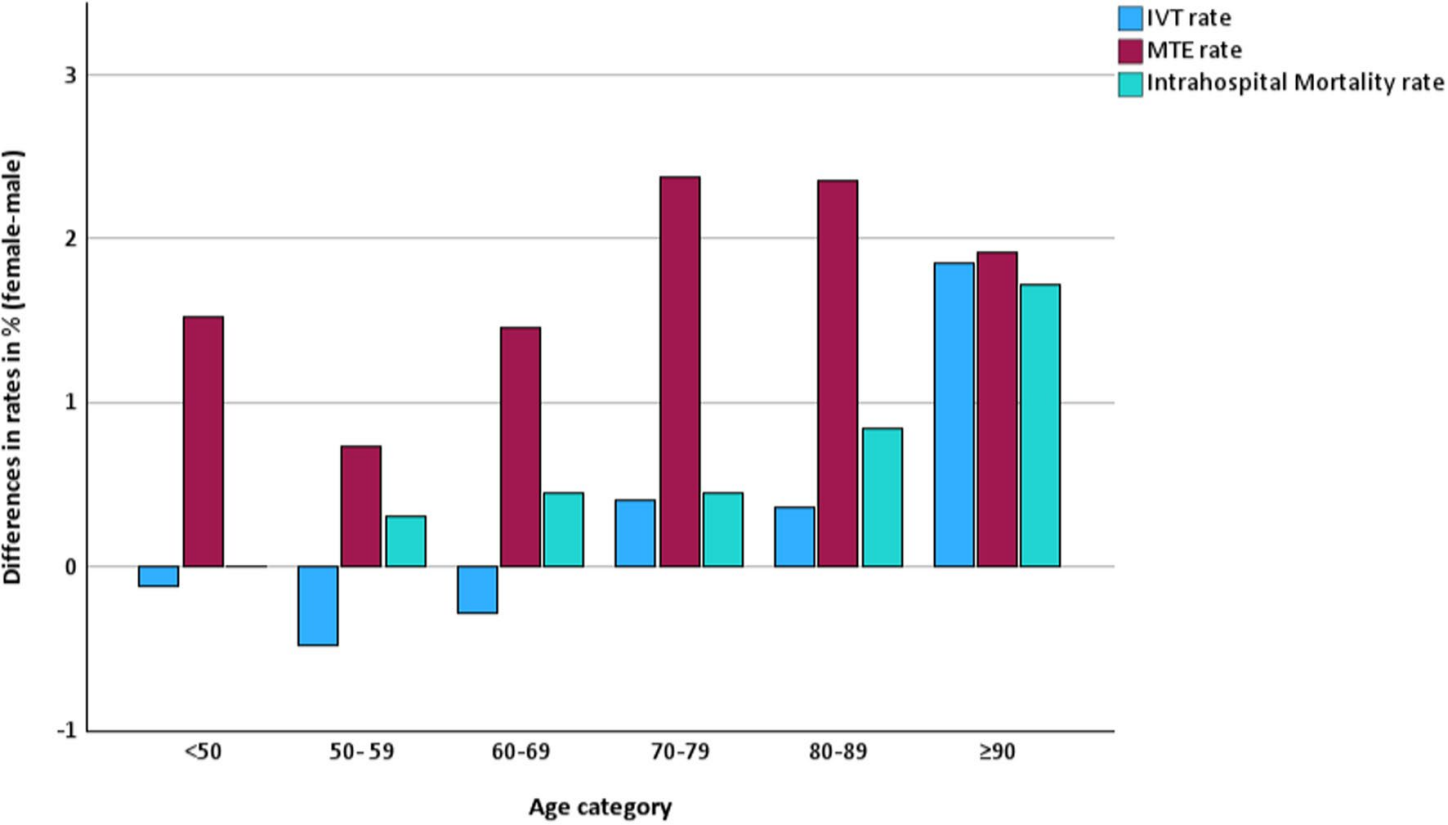
Differences in IVT, MTE, and intrahospital mortality rates for female vs. male patients (2017 to 2022). IVT, intravenous thrombolysis; MTE, mechanical thrombectomy

**Fig. 4:**
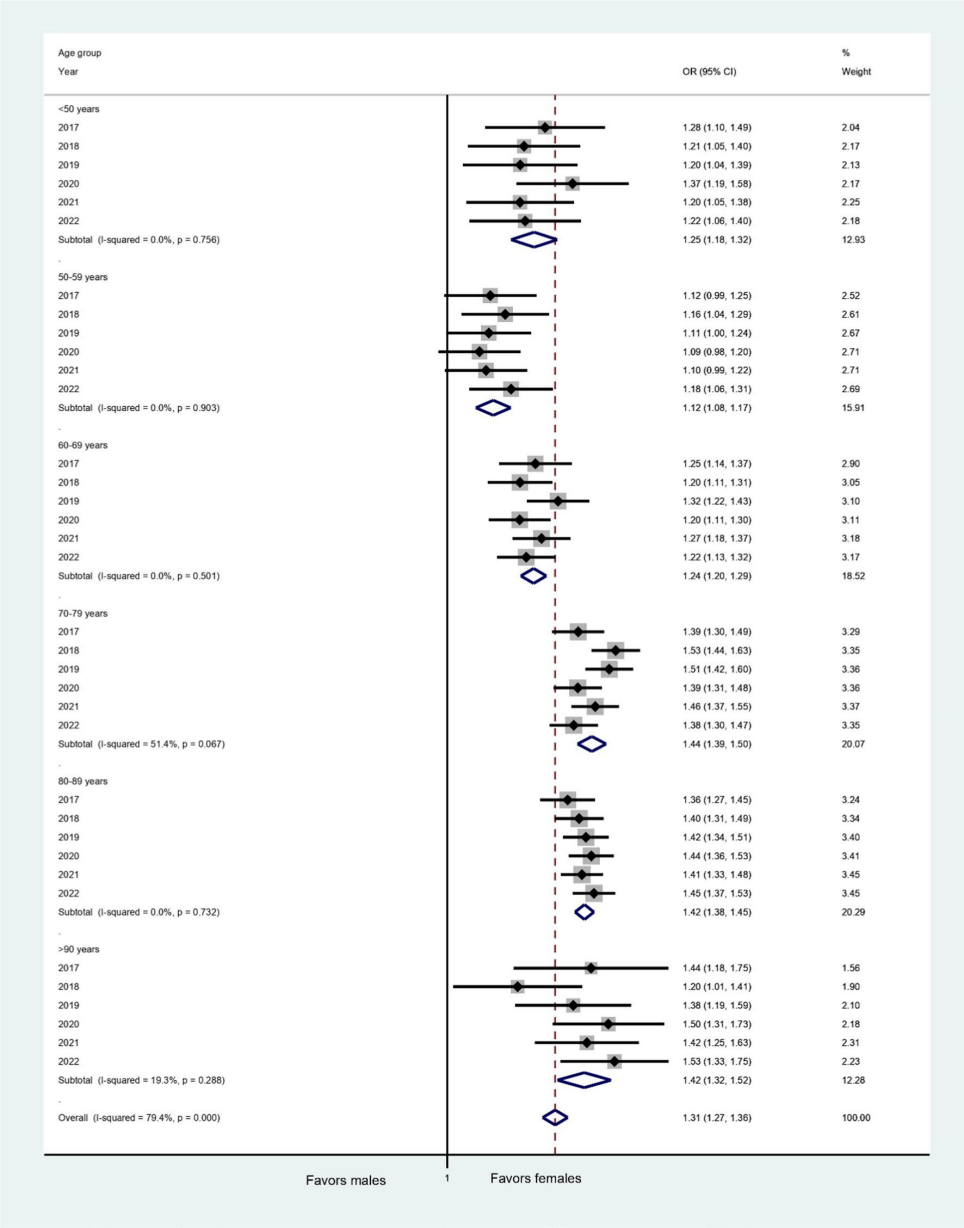
Forest Plot showing yearly ORs for MTE for female vs. male patients. CI, confidence interval; MTE, mechanical thrombectomy; OR, odds ratio

From 2017 to 2022, 215,808 patients were treated with IVT, representing a rate of 16.3% of all patients, with no differences in rates between sexes (16.3% versus 16.3%; *p* = 0.34). We did not find any relevant yearly trend (see Fig. 2a). When adjusted for age categories, female patients appeared to receive IVT more frequently than males, although the difference was small (adjusted OR 1.02 [1.01–1.03]; *p* < 0.01, see Fig. S1). The differences in rates for IVT, MTE, and intrahospital mortality by age category are shown in Fig. 3. The yearly overall IVT rates were stable during the observed period, whereas the MTE rates increased from 5.3% in 2017 to 8.2% (*p* < 0.01) in 2022. The overall MTE rate from 2017 to 2022 was 7.2%. Female stroke patients had a consistently higher MTE rate, even in age-adjusted analysis (8.2% versus 6.3%; *p* < 0.01; adjusted OR 1.34 [CI 1.33–1.36]; *p* < 0.01, see Fig. 2b). The difference in MTE rates between female and male patients increased slightly from 2017 (1.2%) to 2022 (2.3%). The yearly ORs by age category are shown in Fig. 4. The overall treatment rate in SUs was 74.9% (2017–2022). Female ischemic stroke patients had a lower rate of SU treatment (73.6% versus 76.0%; *p* < 0.01). Both trends were consistent on a yearly basis, with a decrease in the difference in SU treatment rates from 2.9% in 2017 to 1.76% in 2022. The lower odds for SU treatment for females were confirmed when adjusted for age categories (adjusted OR 0.92 [CI 0.92–0.93]; *p* < 0.01; see Fig. S2). Admission rates to ICUs were similar for female and male stroke patients in the period of 2020–2022 and slightly greater for females in regression analysis adjusted for age categories (10.6% versus 10.5%; *p* = 0.12; adjusted OR 1.03 [1.02–1.05], *p* < 0.01). We found that treatment rates in early rehabilitation facilities among patients who were not treated in either a SU or ICU were higher in females (10.7% versus 8.6%; *p* < 0.01; adjusted OR 1.10 [1.09–1.12], *p* < 0.01).

### Comparison of intrahospital mortality rates

The intrahospital mortality rate from 2017–2022 was 7.6% for all stroke patients and was significantly higher in female patients (9.1% versus 6.2%; *p* < 0.01). We found similar differences when adjusting for age categories (adjusted OR 1.10 [CI 1.08–1.11]; *p* < 0.01). Female patients who did not survive hospital treatment were generally older (83.9 ± 7.0 vs. 79.0 ± 6.0). Higher mortality rates in females were also consistent every year, and the difference in mortality rates was greatest in the > 90-year-old group (1.8%). Mortality rates in patients who received IVT (8.5% versus 6.0%), MTE (20.8% versus 18.3%), and SU treatment (6.1% versus 4.2%) were also higher in female stroke patients in the years 2017–2022. Similar results were also found when examining subgroups of patients who received bridging thrombolysis and those who were treated with MTE exclusively without IVT from 2020 to 2022.

### Comparison of the prevalence of atrial fibrillation

AF, as a risk factor for embolic stroke, was coded as a comorbidity in 28.8% of stroke patients from 2017 to 2022. Female patients had a significantly higher AF rate (32.6% versus 25.4%; *p* < 0.01). Females with AF were generally older (82.5 ± 7.0 vs 78.1 ± 6.1). AF rates increased continuously with increasing age categories in both female and male patients. The AF rate was also higher in females when adjusted for age categories (adjusted OR 1.05 [CI 1.04–1.05]; *p* < 0.01). The difference in the prevalence of AF in stroke patients according to sex was consistent in the yearly data and decreased from 7.8% in 2017 to 6.5% in 2022. Our analysis revealed that AF prevalence was generally higher among older patients, with the likelihood of AF increasing with patient age. Interestingly, our findings showed that males were more likely to have AF than females only in the age category of patients under 60 years and over 90 years old, whereas the female AF rate was higher in the age categories between 60 and 90 years. Throughout 2017–2022, the difference in AF rates was 7.1%, with higher rates reported in females, and this difference was even greater, by 12.5% (51.8% vs. 39.3%; *p* < 0.01) in patients who had received MTE. In a subgroup analysis, female patients with AF had higher odds of receiving MTE than males when adjusted for age categories (adjusted OR 1.51 [CI 1.48–1.54]; *p* < 0.01). In stroke patients with AF, females also had higher intrahospital mortality rates than males (13.7% versus 10.7%; *p* < 0.01). This result was also consistent for all age categories greater than 50 years (adjusted OR 1.10 [1.08–1.12]; *p* < 0.01).

## Discussion

### Main findings

1. The majority of IS admissions were male, but female patients were older. During the observed period, IVT rates were high and plateaued, while MTE rates continued to increase.
2. Female stroke patients had similar IVT rates versus male patients, while MTE rates were higher for females across all age groups.
3. Female stroke patients had a higher mortality rate during hospitalisation and a lower rate of SU treatment but similar rates of ICU treatment.
4. The prevalence of AF was higher among female stroke patients, and AF was associated with higher intrahospital mortality and MTE rates in females.

### Interpretation and comparison with previous findings

1. Male patients made up the majority of IS patients, as described previously for data from Germany. Globally, including in the United States, the lifetime risk of stroke is estimated to be greater in females with the highest rates being reported in Eastern Europe and Eastern Asia [24]. Sex-specific regional differences in stroke incidence and epidemiology may be attributable to differences in life expectancy and behavioural risk factors [11]. The IVT rates in our study were high and appeared to have reached a ceiling that reflected consistent growth in previous decades. Meanwhile, MTE rates continued to increase. These findings were in line with previously published data from German national datasets [25, 28].
2. Our research revealed that female stroke patients were not at a disadvantage when it came to accessing treatment services, such as IVT and MTE. Other international studies, particularly those that have used pooled and possibly outdated data, have suggested that female stroke patients may be less likely to be treated with IVT [27]. We found that females in Germany were just as likely and, in our age-adjusted analysis, were more likley to receive IVT than their male counterparts. Moreover, we found that women had higher MTE rates. We believe this can be partly explained by the fact that cardioembolic strokes, which lead to more severe strokes and more frequent large vessel occlusions, are more prevalent in women, as evidenced by the higher incidence of AF in women in our study sample [15]. Previous studies have also shown that women are more prone to cardioembolic strokes and strokes caused by valvular heart disease, while men are more frequently affected by strokes caused by large artery atherosclerosis [18]. A recent systematic review found that the rates of MTE were similar in female and male stroke patients globally, with even higher rates found in females in European populations in a regional analysis [21]. The researchers found that studies conducted before 2014 demonstrated lower rates of MTE for female stroke patients. However, studies that used more recent data showed that treatment rates were mostly similar. Higher MTE rates for females in Europe were explained by more frequent cardioembolic strokes as well as a higher capacity for MTE treatments in this region.
3. Our results demonstrated that female stroke patients were consistently at a higher risk for intrahospital mortality. Importantly, the similar IVT rates suggest that differences in the period between the onset of symptoms and presentation at a hospital were not a likely explanation for higher mortality rates, since IVT is the most time-sensitive stroke treatment and can thus be seen as a surrogate for timely presentation. Previous studies have reported that delayed presentations are more common in female patients [20]. Although we found no evidence to suggest that female patients in our study generally had delayed presentations, we cannot exclude the possibility that certain subgroups of female patients may have presented in extended time windows since that type of data was not available in the DRG dataset. Higher intrahospital mortality rates were also found for female stroke patients who were treated with IVT and for MTE across age groups, meaning that this finding was consistent. We believe that the higher mortality rates can be partly explained by higher rates of AF in females, which in turn are associated with a higher risk for embolic strokes and more severe strokes [13, 15].

Older age and more comorbidities are also likely contributors to higher intrahospital mortality rates in females. Previous studies have found that age-adjusted analysis reduced the differences in sex-specific mortality rates [22]. However, when we adjusted for age categories and conducted a subgroup analysis of stroke patients with known AF, we continued to observe higher mortality rates for females in our analysis. This suggests that the effect could not be explained solely by age or AF alone. Similarly, we found that female stroke patients consistently had lower SU treatment rates, whereas ICU treatment rates were similar between female and male stroke patients. Lower SU treatment rates for females have been reported previously in German and Swedish cohorts and were primarily attributed to factors such as delayed presentation, as well as more severe strokes and comorbidities, in female patients [7, 29]. We found that the difference between SU treatment rates (around 2%) could be explained almost entirely by the higher treatment rates in early rehabilitation facilities for female patients who did not receive either SU or ICU treatment. Patients with more severe strokes and comorbidities may have received acute stroke care directly in these early rehabilitation facilities, thereby foregoing SU or ICU treatment altogether. Why a disproportionate number of female patients who did not receive SU or ICU treatment were treated in early rehabilitation facilities remains unclear; however, we assume this is because of the greater age, comorbidities, and stroke severity in the female population.

(4) Our findings showed that when adjusted for age categories, female stroke patients had a higher likelihood of AF. This suggests that more female patients may have experienced embolic strokes, leading to higher MTE rates. AF is one of the main risk factors for embolic strokes and is estimated to increase the overall risk of stroke five-fold [13]. The Rotterdam study demonstrated that the lifetime risk for AF at the age of 55 years was higher in men than in women (23.8% in men and 22.2% in women) and increased with age [12]. Our overall prevalence of AF among ischemic stroke patients was consistent with results from previous studies [9]. Previous findings have suggested that women with AF have a higher risk of embolic stroke, cardiovascular events, and overall mortality associated with AF [2, 5, 10]. Our finding concerning greater MTE rates among female stroke patients with AF further supports the assumption that females with AF are at an increased risk of embolic stroke when compared to males with AF. Previous research has suggested that the increased risk of stroke in women with AF may be due to older age and inequities in cardiovascular care, with more women being undertreated with oral anticoagulation [6]. However, the finding of our subgroup analysis of MTE rates among patients with AF supports the assumption that females with AF are at a higher risk than males of experiencing a severe embolic stroke. Therefore, we believe that the reason underlying the association between higher embolic stroke risk in females with AF is more complex than age or undertreatment alone and requires further research to be fully understood.

### Strengths and limitations

The main strength of our study is the use of a large national dataset of all stroke patients in Germany, thereby minimising the risk of selection bias. Our inclusion of an analysis of the pooled data from 2017 and 2022, an additional sensitivity analysis, age-adjusted ORs, and comparisons of yearly data enabled us to demonstrate that our trends and results were consistent throughout a period of 6 years irrespective of known confounders, further supporting the robustness of our results.

In interpreting our results, several limitations should be considered. We were limited in our analysis by the data available in the Destatis and InEK databases. Data were therefore limited to diagnoses and procedures associated with inpatient treatment services. Patients with in-hospital strokes could not be analysed. We had limited data on patient characteristics as well as outcomes, and no data on long-term outcomes (due to data privacy regulations in Germany). We also could not address possible sex-related differences in outcomes beyond intrahospital mortality rates.

## Conclusion

We found no significant differences in access to stroke treatment services based on sex in Germany. Female patients had similar IVT and higher MTE rates to those of males. However, female stroke patients were less likely to receive SU treatment and had higher intrahospital mortality rates. Greater age and higher prevalence of AF as a surrogate for embolic and severe strokes did not entirely explain these differences. Further research is needed to fully understand sex differences in the epidemiology, outcomes, and mechanisms of stroke.

## Supplementary Information

Additional file 1.

## Data Availability

The datasets used and/or analyzed during the current study are available from the corresponding author on reasonable request.
