# Supplementary material for: No disadvantages for women in acute stroke care in Germany: an analysis of access to stroke treatment services in Germany from 2017 to 2022
Source: Neurol Res Pract. 2025 Feb 20;7:8. doi: 10.1186/s42466-025-00365-4 (PMC11840989; doi:10.1186/s42466-025-00365-4)
Supplement: Supplementary file 1 — Additional file 1. [file 42466_2025_365_MOESM1_ESM.docx]

**Supplemental Materials**

Figure S1: Forrest Plot with yearly ORs for IVT for female vs. male patients


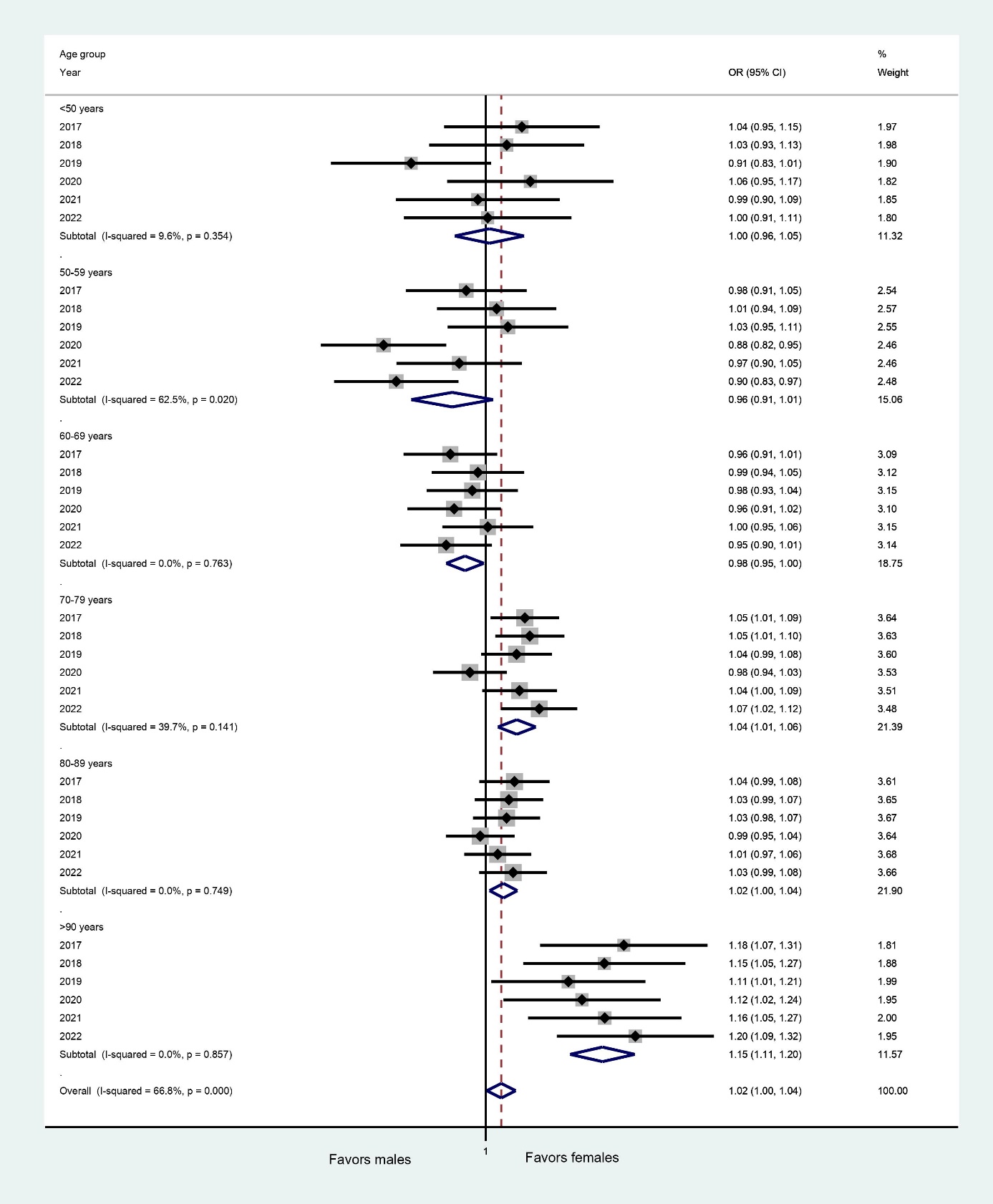


Abbreviations: CI, confidence interval; IVT, intravenous thrombolysis; OR, odds ratio.

Figure S2: Forrest Plot with yearly ORs for SU treatment for female vs. male patients


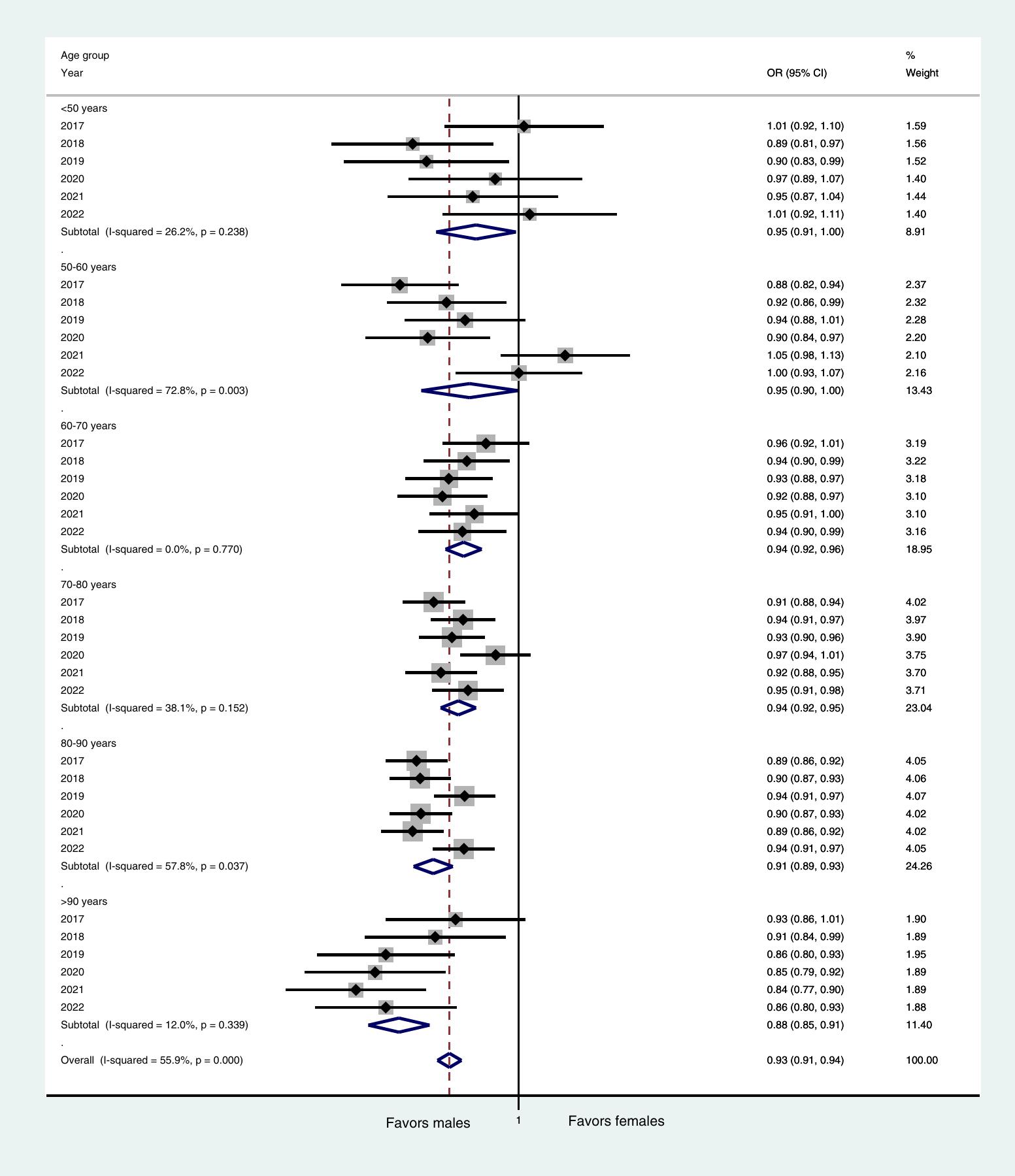


Abbreviations: CI, confidence interval; OR, odds ratio; SU, Stroke Unit.
